# Analgesia for joint punctures in children - experiences with a nitrous oxide / oxygen mixture (LIVOPAN^®^)

**DOI:** 10.1186/1546-0096-9-S1-O37

**Published:** 2011-09-14

**Authors:** Thilo Schmalbach, Gerd Horneff

**Affiliations:** 1 Center for Children Rheumatology, Asklepios Clinic Sankt Augustin, Germany

## Background

Joint punctures in children require adequate analgesia, sedation or both. Suitable for this are intravenous sedatives (eg propofol, midazolam) and analgetics (eg, ketamine and fentanyl). The use of these substances is safe, but requires an intravenous line, monitoring and follow-up. Nitrous oxide/oxygen (N2O/O2) sedation has a long-standing history of safety and success. N2O/O2 has analgic and hypnotic quality characteristics. The mask inhalation of a fixed combination of nitrous oxide with 50% oxygen causes a mean analgesia without loss of consciousness or impairment of protective reflexes for the period of inhalation.

## Methods

We investigated the efficacy and safety of inhaled analgesia in 41 children compared to a control of 102 applications of an intravenous sedation with midazolam and ketamine under the guidelines of the American Academy of Pediatrics (AAP) and the American Society of Anesthesiologists (ASA) for sedation procedures.

## Results

From January 2010 till April 2011 121 children underwent the procedure of a joint puncture in our department. For patients between 7 and 17 years we offered the choice of intravenous sedation with midazolam/ketamin or sedation with Nitrous oxide/oxygen (LIVOPAN^®^) all patients met a ASA 1 or ASA 2 status. In total 41 patients were sedated with LIVOPAN ^®^. 35 applications were successful with good tolerance and adequate analgesia. No patient required a follow up. In 6 cases the procedure was aborted in 4 cases cause of insufficient analgesia, in 2 cases cause by respiratory irritation. After that we did sedation with midazolam and ketamine. 121 patients received sedation with midazolam and ketamine, there were no side effects and the tolerability was excellent. All patients required a follow up on the ward.

## Conclusions

Inhalative analgesia with LIVOPAN^®^ is safe and efficient in short-term interventions with mean intensity of pain. Advantages are the fast and short application, the unneeded intravenous line and the unnecessary follow-up. Disadvantages are not always adequate analgesia and respiratory irritation. The atraumatic application, the early onset of analgesia within 3-5 minutes and the low side effects result in a high level of acceptance of the method in patients, parents and staff. The application of LIVOPAN ^®^ is a good addition and allows a safe and painless joint puncture in older children.

